# Multi-Scale Hydrogen Bonding and Microphase Separation Synergistically Engineered Polyurethane-Polyurea (PU-PUa) as High-Performance Binder

**DOI:** 10.3390/polym18141757

**Published:** 2026-07-18

**Authors:** Hao Wu, Xiaobao Chen, Yi Chi, Weimin Song, Jinyao Li, Zhiqiang Cheng

**Affiliations:** 1 School of Civil Engineering, Central South University, 22 South Shaoshan Rd., Changsha 410075, China; haoutk@csu.edu.cn (H.W.); 224801074@csu.edu.cn (X.C.); wsong8@csu.edu.cn (W.S.); 254801070@csu.edu.cn (J.L.); 2 School of Road and Bridge Engineering, Hunan Communication Polytechnic, Changsha 410132, China; 3 Shanghai Road and Bridge Group Co., Ltd., Shanghai 200433, China; cr420281@163.com

**Keywords:** polyurethane-polyurea (PU-PUa), binder, hydrogen-bonding network, microphase separation, mechanical behavior

## Abstract

Driven by the rising frequency of extreme climatic events and the escalating demand for sustainable infrastructure, modern pavement materials must deliver enhanced resilience, structural stability, and environmental adaptability. This study presents the design and synthesis of a novel polyurethane-polyurea (PU-PUa) pavement binder, engineered via a synergistic framework combining nanoscale microphase separation and a hierarchical hydrogen-bonding network. Utilizing a streamlined, one-step synthesis approach involving an aliphatic isocyanate, a polyaspartic ester, polytetramethylene ether glycol, and 1,4-butanediol, the PU-PUa copolymer achieves distinct nanoscale phase separation between its hard and soft segments. Fourier transform infrared (FTIR) spectroscopy verifies the successful formation of characteristic PU-PUa moieties and a multi-scale hydrogen-bonding network, while DSC and DMA reveal SSC-dependent soft-segment mobility, crystallization/melting behavior, and viscoelastic relaxation. These intra- and inter-segmental interactions, together with thermally activated soft-segment transitions, establish the structural foundation for the macro-performance enhancement of the system. Comprehensive evaluations demonstrate that the PU-PUa binder exhibits excellent mechanical and highly tunable properties. Rheological measurements indicate that increasing the soft segment content (SSC) or incorporating an appropriate diluent concentration significantly lowers the system viscosity, thereby enhancing processing workability during mixing and paving. Contact angle goniometry reveals that the surface hydrophobicity of PU-PUa can be effectively regulated by adjusting the SSC, offering a viable strategy to optimize moisture damage resistance. Moreover, curing behavior analyses show that the polymerization kinetics are strictly governed by both the SSC and environmental temperature, where a lower SSC or elevated curing temperature accelerates strength development. Mechanically, the PU-PUa binder displays desirable surface hardness (>80 Shore A) and exceptional aggregate adhesion (>2 MPa), ensuring robust bonding stability and resistance to traffic-induced abrasion. Characterized by balanced tensile performance, the elongation at break of the binder can be tailored from 90% to 161%, while its tensile strength varies between 6.4 MPa and 17.8 MPa at intermediate temperatures, manifesting excellent resilience and cracking resistance. Overall, this molecular-to-macroscopic design strategy establishes the PU-PUa copolymer as a highly promising, durable binder for next-generation resilient pavement infrastructures.

## 1. Introduction

With the increasing frequency of extreme climate events and a global commitment to sustainable development, it is imperative to enhance the resilience, structural toughness, and environmental safety of pavement materials to facilitate high-quality and sustainable development of highway transportation. Consequently, the development of novel pavement materials that achieve an optimal balance of high strength, elasticity, superior durability, and excellent workability has become a central research priority in road engineering.

Against this background, polymer-based pavement binders have gradually gained traction as promising alternatives to conventional asphalt. Materials such as epoxy resins, polyurethanes, and acrylates are increasingly employed in pavement overlays, bridge deck pavements, rapid repair materials, and porous friction courses due to their tunable mechanical properties, high bonding strength, and improved durability [1,2,3,4,5,6]. Among these, polyurethane (PU) or polyurethane-based binders stand out for their unique advantages: they offer excellent flexibility even at low temperatures, desirable abrasion resistance, fast curing at ambient temperatures, exceptional aggregate adhesion, and the ability to tailor mechanical performance through molecular design [7,8,9,10,11,12]. As a result, PU binders have been widely investigated for applications including pavement cold recycling, ultra-thin wearing courses, and asphalt modification.

However, despite these benefits, conventional PU binders suffer from several critical limitations when applied in pavement engineering. A primary concern is their pronounced moisture sensitivity; the parasitic reaction between isocyanate groups and ambient moisture often triggers foaming and internal porosity, which significantly compromises the structural integrity and durability of the pavement. Moreover, standard PU formulations frequently struggle to balance high-temperature stiffness with low-temperature crack resistance, making them susceptible to premature failure under cyclic traffic loads and extreme temperature fluctuations. Furthermore, most PU systems, especially those based on aromatic isocyanates, are vulnerable to weathering degradation and thermo-oxidative aging, resulting in surface yellowing, chain scission, and loss of mechanical properties over time [13,14,15,16,17]. These deficiencies necessitate the development of advanced molecular engineering strategies to optimize the internal architecture of PU-based materials for more resilient pavement systems.

In this context, polyurethane-polyurea (PU-PUa) copolymers have emerged as a highly promising next-generation binder. By incorporating polyurea (PUa) structures through the reaction of isocyanates with amine-terminated compounds (e.g., polyaspartic esters), the resulting PU-PUa system introduces strong urea linkages (-NHCONH-), which form significantly more robust hydrogen bonds than conventional urethane linkages (-NHCOO-). This unique feature drives a well-defined microphase separation structure with nanoscale hard segment domains acting as physical crosslinks, while the continuous soft segment phase provides excellent elasticity. Compared to conventional PU, PU-PUa offers several distinct advantages [18,19,20]: (1) superior resistance to moisture and hydrolysis due to the higher density of stable hydrogen bonds and lower content of free isocyanate groups; (2) enhanced weatherability and anti-aging performance, especially when using aliphatic isocyanates; (3) tunable curing kinetics and extended pot life via the steric hindrance effect of polyaspartic esters, allowing better on-site constructability; (4) improved workability with lower viscosity at higher soft segment content (SSC); and (5) a unique combination of high strength, exceptional toughness, and elastic recovery, which is essential for withstanding repeated traffic loads and thermal stresses. These attributes make PU-PUa an ideal candidate for demanding pavement applications, including rapid repair materials, high-elasticity noise-reducing surfaces, bridge deck pavements, and durable porous friction courses.

Despite these theoretical advantages, the application of PU-PUa in pavement engineering is still in its nascent stages, with limited understanding of the fundamental correlation between multi-scale hydrogen bonding, microphase morphology, and macroscopic engineering performance. Specifically, the relationships between the molecular architecture (particularly the SSC) [19,20,21], the resulting microphase morphology (from hard-segment continuous to soft-segment continuous), and the macroscopic mechanical performance—especially fracture toughness—have not been systematically elucidated for PU-PUa systems. Most existing studies lack a gradient design that spans the percolation threshold, leaving the optimal composition for balanced strength, ductility, and fracture resistance undiscovered. Furthermore, the regulatory mechanisms by which SSC influences workability, curing kinetics, and the non-monotonic fracture behavior remain poorly understood.

To bridge these critical gaps, this study introduces a rational molecular design and a streamlined one-step synthesis strategy to develop an advanced polyurethane-polyurea (PU-PUa) pavement binder. Distinct from studies focused on conventional PU-PUa synthesis, the core novelty of this work lies in establishing a comprehensive, soft segment content (SSC)-dependent “formulation–structure–performance” framework specifically tailored for pavement engineering. By systematically adjusting the SSC, this research explicitly maps the correlations between the copolymer’s structure and its multi-dimensional macro-engineering behaviors, including rheological evolution, isothermal curing kinetics, surface/interfacial properties, and mechanical performance (encompassing viscosity trajectory, contact angle, aggregate bonding strength, failure modes, hardness, tensile profiles, and resilient modulus). Furthermore, Fourier transform infrared (FTIR) spectroscopy and atomic force microscopy (AFM) are integrated to deconstruct the underlying chemical moieties and surface micro-morphologies, providing robust, multi-scale evidence to validate the proposed structure-property mechanisms. Ultimately, these findings clarify the cooperative mechanisms by which segmental composition and hydrogen-bonding networks govern binder performance, thereby providing a rigorous scientific blueprint for engineering highly workable, high-performance PU-PUa binders for next-generation resilient infrastructures.

## 2. Materials

The material design strategically of PU-PUa incorporates an aliphatic isocyanate (HDI trimer) as the crosslinking core of the hard segments to provide rigid support, a polyether diol as the soft segment to impart flexibility, and polyaspartic ester as an amine-based chain extender that works synergistically with BDO to form a hard segment microstructure characterized by strong hydrogen bonding and high structural regularity. This design deliberately induces a well-defined microphase separation structure. The fundamental properties of raw materials are listed in Table 1.

### 2.1. Aliphatic Isocyanate

**Figure 1:**
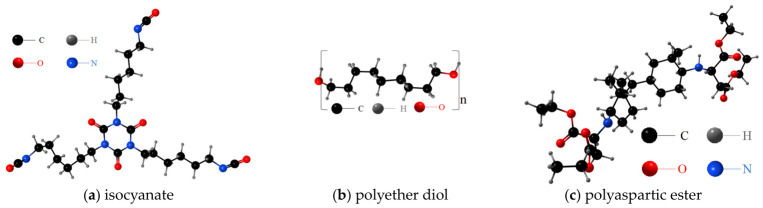
Schematic diagram of isocyanate, polyether diol and polyaspartic ester.

A trifunctional aliphatic isocyanate with a cyclic molecular architecture, specifically, the trimer of hexamethylene diisocyanate (HDI trimer), supplied by Wanhua Chemical Group Co., Ltd. in Yantai, China, was employed as the hard-segment precursor. This configuration provides high crosslinking density and rigid nodes, which facilitate the ordered aggregation of hard segments and promote distinct microphase separation. Such structural features are essential for optimizing the binder’s mechanical modulus, tensile strength, and thermal stability. The chemical structure of the isocyanate is illustrated in Figure 1a.

### 2.2. Polyether Diol

Polyether diol, specifically polytetramethylene ether glycol, supplied by Lido Chemical Co., Ltd. in Jining, China, was selected to constitute the soft segments due to its intrinsic hydrolysis resistance and backbone flexibility, which are critical for pavement durability in humid and low-temperature environments. Compared to polyester-based diols, this polyether-type precursor offers superior hydrolytic stability and remains liquid at room temperature, ensuring better processing workability. Its chemical structure is presented in Figure 1b.

### 2.3. Polyaspartic Ester

The secondary chain extender used was a polyaspartic ester, a sterically hindered aliphatic secondary diamine synthesized from the Michael addition reaction of maleic acid with an aliphatic primary diamine, supplied by Wei’er Level Ground Materials Co., Ltd. in Shenzhen, China. The significant steric hindrance of this diamine regulates the reaction kinetics with isocyanates, effectively extending the pot life (working time) for field applications compared to primary amines. The chemical structure of polyaspartic ester is shown in Figure 1c.

The fundamental properties of the main materials for the synthesis of PU-PUa copolymer are summarized in Table 1.

### 2.4. Additives

1,4-butanediol (BDO), supplied by Wanhua Chemical Group Co., Ltd. in Yantai, China, was employed as a low-molecular-weight diol chain extender. Its lower reactivity compared to amine-based extenders allows for a more controlled polymerization process, while its linear structure facilitates the structural regularity of the hard-segment domains. To ensure the integrity of the cast specimens, a silicone-based defoaming agent (BYK 066N) was utilized to effectively eliminate micro-bubbles and prevent air entrapment during high-speed agitation, thereby minimizing internal defects and ensuring the reliability of mechanical characterizations. Additionally, ethyl acetate served as a diluent to optimize the system’s rheological behavior and promote the homogeneous dispersion of resins and additives, ensuring the formation of a uniform crosslinked network. Moreover, if needed, resin-free color paste can also be added in the mixture for color adjustment.

### 2.5. Mix Proportions for PU-PUa Copolymer

The PU-PUa developed in this study is intended for pavement materials, requiring both adequate mechanical performance and a sufficient workability. The formulation of PU-PUa was developed through a systematic empirical approach. First, exploratory tests and single-factor experiments identified viable dosage ranges for each component: isocyanate (30–45%), polyaspartic ester (40–55%), polyether diol (10–28%), and BDO (0–8%). In this study, the NCO index, defined as n(NCO)/[n(OH)+n(NH)], was set at 1.0; the mass of each component was calculated from the NCO equivalent, NH equivalent, and OH equivalent values together with the molar ratios presented in Table 2. Within these ranges, five representative formulations were selected to create a gradient of SSC (Table 2), enabling systematic investigation of the influence of SSC on macroscopic properties.

The design of these five formulations follows two key principles. First, the SSC is defined as the weight fraction of polyether diol relative to the total reactive components. SSC increases from 14% to 26% across Groups I to V, providing a formulation range for investigating the gradual shift in mechanical response from hard-segment-dominated behavior toward soft-segment-dominated behavior. This range intentionally spans the percolation threshold of hard segment microdomains, where the mechanical response shifts from rigid and high-strength to flexible and tough, allowing identification of an optimal balance for pavement applications.

Second, as the SSC increases, the molar ratios of isocyanate, polyaspartic ester, and BDO are simultaneously reduced. This coordinated decrease serves three purposes: (i) to maintain the overall stoichiometric balance between –NCO and reactive hydrogens (–OH from polyether diol and BDO, –NH from polyaspartic ester), ensuring complete reaction and minimizing residual isocyanate; (ii) to systematically dilute the density of urea and urethane linkages, thereby attenuating the physical crosslinking density of hard segment domains; and (iii) to avoid abrupt changes in the hard/soft segment interface, preserving a well-defined microphase separation structure across the composition range. In particular, the BDO content first increases from 1.11 (14% of SSC) to 1.54 (17% of SSC) and then gradually decreases to 0.40 (26% of SSC). This non-monotonic trend reflects an optimization of chain extension efficiency: at low of SSC, higher BDO helps extend hard segments and reinforce the rigid skeleton; at high SSC, excessive BDO would cause over-crosslinking and impair flexibility. Hence, the BDO variation is tuned to maintain the structural integrity of hard microdomains without sacrificing the compliance of the soft matrix.

### 2.6. Synthesis Mechanism of PU-PUa Copolymer

#### 2.6.1. Synthesis of PU-PUa Copolymer

In this study, a one-step synthesis protocol was adopted to fabricate the PU-PUa copolymer. According to the designed mix proportions, polyaspartic ester, BDO, and the BYK defoaming agent were first premixed uniformly and kept for subsequent use. Then, the isocyanate and polyether diol were stirred at 1000 rpm for 3 min to ensure adequate dispersion. Immediately afterward, the premixed polyaspartic ester/BDO/BYK component was added to the isocyanate/polyether diol mixture, and the whole system was further stirred at 1000 rpm for 3 min. After stirring, the mixture was allowed to stand for 3 min to release entrapped bubbles and was then poured into molds to prepare specimens. The specimens were demolded after 6 h and subsequently cured at 25 °C until the designated testing age. No separate prepolymerization step was conducted during this process.

Unlike the multi-stage prepolymer method, this approach involves the synchronous introduction of all reactive components, where the final elastomer architecture is governed by the differential nucleophilic reactivity of various hydrogen-containing functional groups toward the isocyanate. This “in situ” assembly facilitates the simultaneous evolution of a multi-scale hydrogen-bonding network and a nanoscale microphase-separated morphology. From an engineering perspective, this streamlined workflow significantly reduces processing complexity, energy consumption, and labor intensity, while the meticulous molecular design ensures that the resulting binder maintains superior structural integrity and mechanical performance tailored for pavement applications.

#### 2.6.2. Copolymerization Mechanism of PU-PUa

The synthesis of PU-PUa involves a competitive polyaddition process. The synthesis of PU is typically performed through a polyaddition reaction between isocyanates (-NCO) and polyols. In contrast, PUa is synthesized via a polyaddition reaction involving isocyanates (-NCO) and polyamines [19,20].

PU: R-NCO + R′-OH → R-NH-COO-R′(1)

PUa: R-NCO + R″-NH_2_ → R-NH-CO-NH-R″ (2)

When the precursor materials for PU and PUa (isocyanate, polyol, and amine compounds) are combined, the isocyanate group (-NCO) undergoes simultaneous reactions with both hydroxyl (-OH) and amine (-NH_2_) groups, resulting in the formation of a PU-PUa copolymer. Therefore, the final product of the reactions is characterized by the presence of both urethane linkages (-NHCOO-) and urea linkages (-NHCONH-), forming a complex and cross-linked polymer network. The formation of PU-PUa copolymer can be represented as:

PU-PUa: [-NH-COO-R′-]*_x_* [-NH-CO-NH-R″-]*_y_* (3)

where *x* and *y* denote the molar repetitions of the PU and PUa segments, respectively.

The core feature of the one-step process is the simultaneous or sequential occurrence of multiple competing reactions. Polyaspartic ester, a sterically hindered secondary amine, exhibits significantly higher reactivity than alcohols. During the initial mixing stage, the -NCO groups preferentially react with the -NH- groups of polyaspartic ester, generating hard segments containing highly polar urea bonds. This step is crucial for constructing a robust hydrogen bonding network and hard segment microstructure. Subsequently, BDO (a small-molecule primary alcohol) reacts with the isocyanate, further extending the hard segments. The primary hydroxyl groups of polyether diol also participate, connecting the flexible soft segment chains to the growing polymer network.

Due to the higher reaction priority of polyaspartic ester and BDO, hard segments composed of urea and urethane bonds are generated in situ. Driven by strong intermolecular forces (e.g., hydrogen bonding), these hard segments immediately aggregate upon formation. Unlike the prepolymer method—where the soft segment backbone is first synthesized and then hard segments are attached—the one-step process involves nearly simultaneous hard segment generation and microphase separation. As hard segment chains elongate, the system spontaneously undergoes nanoscale phase separation to reduce mixing enthalpy with the flexible polyether diol soft segments, forming hard segment microdomains dispersed within the soft segment matrix.

The steric hindrance of polyaspartic ester moderately retards urea bond formation, providing relaxation time for molecular chains to pack more orderly, thereby forming regular and effective hard segment microdomains. As a typical block copolymer, the PU-PUa molecular chains consist of alternating hard and soft segments connected by covalent bonds [21]. This chemical structure induces thermodynamic incompatibility between the segments, leading to a microphase separation structure—a key factor determining the mechanical properties of PU-PUa.

#### 2.6.3. Molecular Composition of PU-PUa Copolymer

**Figure 2:**
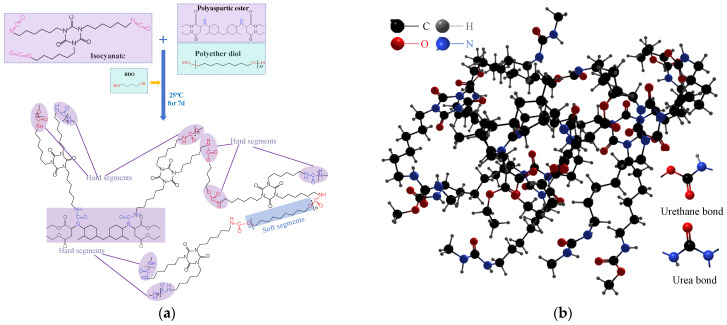
Molecular composition of PU-PUa copolymer. (**a**) molecular structures for synthesis of PU-Pua. (**b**) idealized PU-PUa structure.

The molecular structure of the PU-PUa copolymer is rationally assembled from flexible long-chain polyether diols (soft segments), rigid isocyanate-derived moieties, and sterically hindered polyaspartic ester segments (hard segments). The molecular structure for the synthesis of PU-PUa and the resulting idealized PU-PUa structure are illustrated in Figure 2.

The urethane linkage (-NHCOO-) serves as the fundamental repeating unit of the PU component. In this segmented block copolymer, the hard segments—formed by the polyaddition of diisocyanates and BDO—exhibit high polarity, which fosters an extensive network of inter-segmental hydrogen bonds. While the majority of these interactions occur within the hard domains to provide structural reinforcement, a secondary population of hydrogen bonds forms between the hard and soft segments. This hierarchical hydrogen-bonding network significantly enhances intermolecular cohesion, thereby dictating the material’s macroscopic strength and elastic recovery.

Complementing the PU domains, the PUa segments are defined by the urea linkage (-NHCONH-). Compared to the asymmetric urethane group, the urea bond possesses a geometrically symmetric configuration and a higher density of hydrogen-bond donors (two NH groups per carbonyl). This bidentate hydrogen-bonding capability facilitates a significantly higher physical crosslinking density and thermodynamic stability. Therefore, the incorporation of PUa segments imparts superior environmental resilience to the binder, including enhanced resistance to thermo-oxidative aging, chemical ingress, and abrasive wear. Furthermore, the strong electron-donating effect of the nitrogen atoms in the urea group accelerates the reaction kinetics, enabling rapid, catalyst-free curing—a critical advantage for on-site pavement construction.

## 3. Experimental Methods

### 3.1. Chemical and Microstructural Characterization

The chemical structure of PU-PUa was characterized using Fourier transform infrared (FTIR) spectroscopy equipped with an attenuated total reflection (ATR) accessory. Atomic force microscopy (AFM) was used to observe the surface morphology and provide qualitative morphological information related to the proposed segmented structure of PU-PUa. Differential scanning calorimetry (DSC) and dynamic mechanical analysis (DMA) were performed to evaluate thermal transitions, soft segment crystallization behavior, and temperature-dependent viscoelastic properties.

### 3.2. Processing Characterization

The processing characteristics including viscosity and curing of the PU-PUa binder was systematically investigated by analyzing its transition from a liquid emulsion to a solid elastomer. This analysis allows for a comprehensive quantification of the binder’s “working window” and its temperature-dependent strength development, which are critical for optimizing on-site construction protocols.

The dynamic viscosity of the PU-PUa binder was characterized using a rotational viscometer to assess its suitability for field applications. Measurements were conducted at 25 °C utilizing a No. 27 spindle. The shear rate was varied within a range of 15 to 60 rpm to maintain torque readings between 10% and 98%, ensuring the linearity and accuracy of the rheological data. This evaluation provides critical insights into the binder’s workability and its ability to sufficiently coat aggregate surfaces.

To further elucidate the influence of environmental conditions on the polymerization process, the viscosity evolution of the binder was monitored over time. Standard dumbbell-shaped specimens were initially cast and pre-cured at 25 °C for 24 h. Subsequently, isothermal curing was performed in constant-temperature chambers at 15 °C, 25 °C, 35 °C, 45 °C, and 60 °C. Six replicates were prepared for each temperature group to quantify the relationship between thermal energy and the strength development rate of the PU-PUa system.

### 3.3. Interfacial Properties Between Binder and Aggregate

The hydrophobic and interfacial properties of PU-PUa binder and aggregate were evaluated through wettability, bonding strength, and modified boiling tests under dry and wet conditions. By correlating hydrophobic and interfacial properties with bonding performance, the compatibility of the system and its capacity to maintain long-term structural integrity in the presence of aggregate-binder interfaces were elucidated.

#### 3.3.1. Wettability Test

The hydrophobic property of the PU-PUa were evaluated through static water contact angle (WCA) measurements. Deionized water droplets were deposited onto the specimen surface, and the contact angles were determined using a high-precision goniometer. To ensure statistical reliability, the average WCA was calculated from measurements taken at three distinct locations on each specimen.

#### 3.3.2. Interfacial Bonding Strength Test

The interfacial bonding strength between the binder and aggregate was evaluated using a pull-off test in accordance with ASTM D4541. The binder was uniformly applied onto the substrate surface, and then the pull-off dollies were placed onto the binder coating. A copper wire with a diameter of 0.2 mm was employed to control the thickness of the binder film between the dolly and the substrate. After the specimens had fully cured, the pull-off test was conducted, and the maximum pull-off force was recorded to calculate the bonding strength at the binder-substrate interface. Each test configuration was tested in triplicate to obtain the average interfacial bond strength.

#### 3.3.3. Modified Boiling Test

The water stability of conventional polyurethane binders has always been a major concern when they are utilized as pavement binders, as their poor resistance to moisture attack can readily induce various distresses such as raveling and stripping in pavements. In this study, a modified boiling test based on ASTM D3625 was adopted to evaluate the adhesion state between the binder and aggregate under hydrothermal conditions.

First, aggregate particles with a size range of 9.5–13.2 mm were washed with deionized water to remove surface dust, then dried in an oven at 105 ± 5 °C to constant weight, and cooled to room temperature for subsequent use. Prior to coating, a copper wire was tightly wrapped around each aggregate particle. The aggregate was then completely immersed in the polymer or asphalt emulsion to ensure full coverage of its surface. The specimen was initially placed in gently boiling water for 3 min. Subsequently, the copper wire was withdrawn from the binder film on the specimen surface to create a prefabricated defect, thereby introducing a controlled circumferential weak interface within the cured binder film. This prefabricated defect allows peeling to initiate preferentially from this weakened region upon water exposure. Such a design enables a more reliable assessment of the intrinsic debonding resistance between the binder film and the aggregate surface.

After creating the defect, the specimens were further immersed in boiling water for another 10 min. Following the boiling treatment, the peeling behavior of the cured polymer or asphalt film from the aggregate surface was observed. Special attention was paid to whether peeling preferentially initiated at the prefabricated defects, thereby evaluating the interfacial stripping resistance between the polymer film and the aggregate.

### 3.4. Mechanical Performance Investigation

The mechanical integrity of the PU-PUa system was characterized through surface indentation and uniaxial tensile tests. This combined evaluation of hardness and tensile behavior provides a holistic view of the binder’s structural stability, specifically its ability to balance high-load bearing capacity with the elastic ductility required to accommodate traffic-induced strains.

The surface resistance to indentation was measured using a Shore durometer. The specimens were placed on a rigid, level surface, and the hardness was recorded after the indenter maintained stable contact with the material. Three measurements were performed per sample to ensure consistency in the crosslinking density across the specimen surface. The mechanical strength and ductility of the PU-PUa binder were characterized through uniaxial tensile testing following ASTM D412. Dumbbell-shaped specimens were loaded to failure using a universal testing machine at a constant displacement rate of 50 mm/min. The tensile strength and elongation at break were recorded to evaluate the material’s ability to withstand traffic-induced strain and resist fracture.

## 4. Results and Discussion

### 4.1. Chemical and Microstructural Characterization of PU-PUa Copolymer

#### 4.1.1. FTIR Analysis

**Figure 3:**
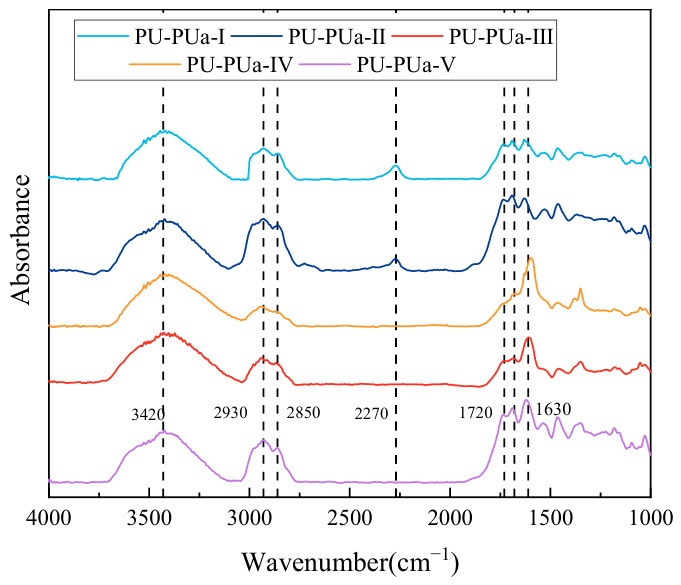
FTIR spectra of PU-PUa.

Fourier transform infrared spectroscopy (FTIR) was employed to identify the characteristic absorption peaks and provide chemical structural information relevant to the PU-PUa system, as shown in Figure 3. The broad absorption band around 3420 cm^−1^ is mainly assigned to hydrogen-bonded N-H stretching vibrations associated with urethane and urea groups. Before ATR-FTIR testing, the specimens were vacuum-dried for 30 min and measured under dry conditions to minimize the influence of absorbed moisture. Nevertheless, a minor contribution from residual O-H stretching vibrations cannot be completely excluded. The peaks at 2930 cm^−1^ and 2850 cm^−1^ correspond to the asymmetric and symmetric stretching vibrations of CH_2_, primarily originated from the methylene groups in polyether diol soft segments, supporting the successful incorporation of the polyether soft segments into the PU-PUa network. 

The characteristic absorption peak of isocyanate groups (-NCO) near 2270 cm^−1^ was not detected, indicating that the -NCO groups were largely consumed during the reaction and that no obvious free -NCO remained within the detection limit of FTIR. In the carbonyl (C=O) region, the distinct peak at 1720 cm^−1^ corresponds to the stretching of free urethane carbonyls within the PU domains, whereas, the intense absorption at 1630 cm^−1^ is attributed to the stretching of urea carbonyls in the PUa segments. The relatively lower wavenumber of the urea peak compared to the urethane peak highlights the stronger hydrogen-bonding capability of the urea linkages, which underpins the superior structural stability of the PUa-rich hard domains. It should be noted that FTIR confirms the formation of urethane and urea groups and provides evidence of hydrogen-bonding interactions, but it does not directly demonstrate the spatial distribution of hard and soft segments or the formation of bulk microphase separation.

#### 4.1.2. AFM Analysis

**Figure 4:**
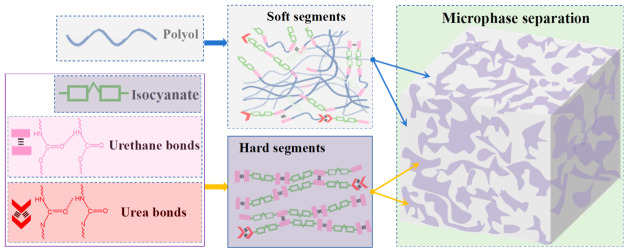
Proposed schematic illustration of the segmented structure of PU-PUa.

Figure 4 presents a schematic diagram illustrating the possible molecular arrangement of soft and hard segments in the PU-PUa system. Based on the segmented molecular architecture and intermolecular hydrogen-bonding interactions, the hard segments are expected to aggregate and act as nanoscale physical crosslinking domains within the soft-segment-rich matrix. This schematic is intended to describe the proposed structural model rather than serve as direct experimental evidence of microphase separation.

**Figure 5:**
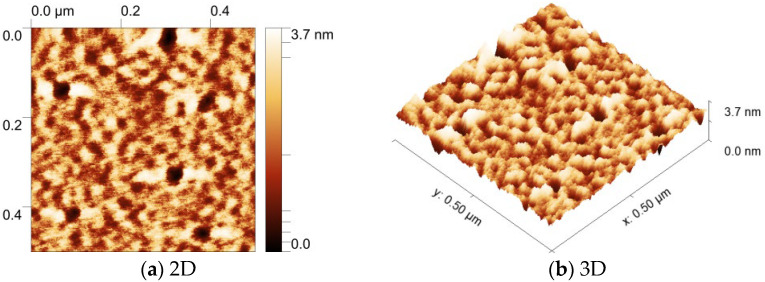
Morphology of PU-PUa.

Atomic force microscopy (AFM) was used to observe the surface morphology of PU-PUa. Figure 5 showed AFM height images of the PU-PUa sample over a scan area of 0.5 μm × 0.5 μm. The images reveal obvious surface topographical heterogeneity, which is consistent with the proposed segmented morphology of the PU-PUa system. The bright, elevated regions may be associated with relatively rigid hard-segment-rich domains, while the darker, recessed regions may correspond to softer polyether-rich regions. The observed topographical contrast may arise from differences in local composition and/or mechanical response between these regions. These hard-segment-rich domains are expected to act as physical crosslinking points, contributing to structural strength and deformation resistance. Meanwhile, the soft-segment-rich regions are expected to provide flexibility and elastic energy dissipation. Therefore, the AFM observations support the proposed structure-property interpretation of PU-PUa, in which hard-segment-rich domains contribute to strength, while soft-segment-rich regions contribute to flexibility. However, AFM primarily provides qualitative surface morphological information; it does not directly quantify the bulk microphase separation, domain size distribution, or phase continuity of the PU-PUa system.

#### 4.1.3. DSC Analysis

**Figure 6:**
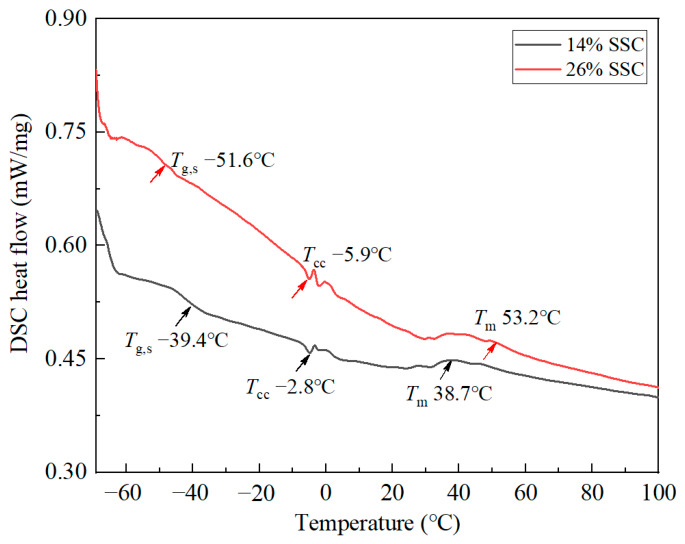
DSC heat flow curves of PU-PUa with various SSC.

To elucidate the influence of SSC on the thermal transitions and microstructural evolution of the PU-PUa system, differential scanning calorimetry (DSC) was performed on representative formulations with 14% and 26% SSC. The second-heating DSC curves, recorded over a temperature range of −70 °C to 100 °C at a heating/cooling rate of 10 °C/min with an isothermal hold at −70 °C for 5 min to ensure a consistent thermal history, are presented in Figure 6. The corresponding thermal parameters extracted from the DSC thermograms are summarized in Table 3.

As shown in Table 3, increasing the SSC from 14% to 26% significantly lowered the glass transition temperature of the soft segments (*T*_g,s_) from −39.4 to −51.6 °C. This pronounced depression of *T*_g,s_ indicates substantially enhanced segmental mobility of the polyether diol chains, arising from the reduced physical constraints imposed by the hard domains and the hydrogen-bonded network. The diminished confinement at higher SSC promotes a more relaxed state of the soft-segment-rich phase, which is a characteristic of enhanced microphase separation between the hard and soft segments.

In addition to the glass transition, both samples exhibited a weak exothermic peak near −5 °C, which is attributed to cold crystallization or crystal reorganization of the soft segments of polyether diol during heating. The cold crystallization enthalpy (Δ*H*_cc_) for the 26% SSC sample (0.173 J/g) was markedly higher than that for the 14% SSC sample (0.089 J/g), indicating that the higher SSC system possesses greater chain mobility and a stronger tendency toward chain rearrangement and ordering. This observation suggests that increasing the SSC facilitates the formation of more organized soft-segment structures, even though the overall crystallinity remains low.

The endothermic peaks observed at elevated temperatures correspond to the melting of polyether diol crystalline domains formed during the heating process. The apparent melting temperature (*T*_m_) increased from 38.7 °C for the 14% SSC sample to 53.2 °C for the 26% SSC sample, while the melting enthalpy (Δ*H*_m_) decreased from 2.119 J/g to 0.414 J/g. This seemingly contrasting behavior, a rise in melting temperature accompanied by a reduction in enthalpy, is consistent with the formation of a smaller population of more perfect, thermally stable crystallites at higher SSC. The increased chain mobility at higher SSC allows polyether diol segments to pack more efficiently, yielding crystallites with higher lamellar thickness and thermal stability, albeit at a lower overall crystalline fraction due to the dilution effect of the hard segments.

Overall, the systematic variations in *T*_g,s_, Δ*H*_cc_, *T_m_*, and Δ*H*_m_ demonstrate that increasing the SSC (1) enhances the chain mobility of the soft segments of polyether diol, (2) facilitates cold crystallization and structural reorganization, and (3) promotes the formation of thermally more stable soft-segment crystalline domains. These findings underscore the critical role of SSC in governing the microphase-separated morphology and thermal properties of the PU-PUa system, and provide a thermodynamic basis for understanding the subsequent mechanical behavior, where the balance between hard-domain reinforcement and soft-segment flexibility dictates the overall performance of the binder.

#### 4.1.4. DMA Analysis

**Figure 7:**
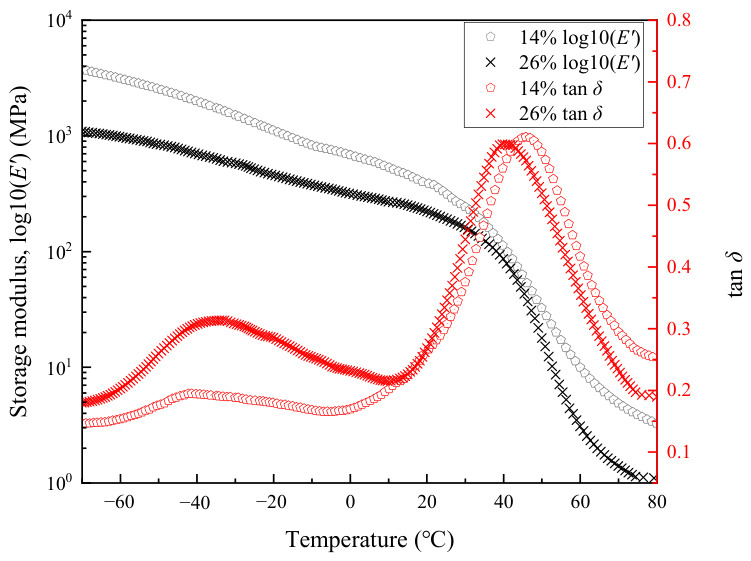
DMA results of PU-PUa with various SSC as functions of temperature.

Dynamic mechanical analysis (DMA) was employed to probe the viscoelastic response of PU-PUa with contrasting SSC (14% and 26%), providing insights into the temperature-dependent segmental dynamics and their correlation with the underlying microphase-separated morphology. Figure 7 presents the storage modulus (*E*′) and loss factor (tan *δ*) as functions of temperature over the range from −70 °C to 80 °C. Throughout the entire temperature window, the 14% SSC PU-PUa exhibited consistently higher *E*′ values than its 26% SSC counterpart. This disparity indicates that at lower SSC, the higher volumetric fraction of hard segments, reinforced by dense urea/urethane hydrogen-bonding associations, imposes stronger physical constraints on chain mobility, thereby enhancing the material’s elastic energy storage capacity and overall rigidity. In contrast, the elevated SSC system manifests significantly reduced *E*′ across all temperature regimes, reflecting the enhanced chain flexibility of the soft segments of polyether diol and the attenuated hard-domain connectivity. This observation is consistent with a transition toward a soft-segment-continuous morphology, where the elastic response is increasingly governed by entropic elasticity rather than by a rigid percolating hard-phase network.

Both formulations exhibit two distinct relaxation processes in the tan–tan *δ* spectra: a low-temperature peak and an intermediate-to-high-temperature peak. The low-temperature tan *δ* peak is assigned to the glass-transition-related relaxation (α-relaxation) of the soft segments of polyether diol, commonly referred to as the dynamic mechanical glass transition temperature (*T*_α_). For the 14% SSC sample, the *T_α_* appears at approximately −42.6 °C, which is in reasonable agreement with its DSC-derived *T*_g,s_ (−39.4 °C), confirming that this peak primarily originates from the cooperative segmental motion of the soft-segment-rich phase. For the 26% SSC sample, however, the *T*_α_ is observed at approximately −41.4 °C, which is significantly higher than its DSC *T*_g,s_ (−51.6 °C). This discrepancy arises from the difference in the nature of the two techniques: DSC measures the thermodynamic glass transition under quasi-static heating, whereas DMA probes the kinetic, frequency-dependent relaxation under oscillatory mechanical loading. In the 26% SSC system, the reduced hard-domain constraint allows a broader distribution of relaxation times, and the DMA peak reflects not only the average segmental mobility but also the cooperative rearrangements of soft segments interacting with the soft–hard interfaces. Therefore, the tan *δ* peak temperature should be interpreted as a dynamic mechanical relaxation indicator rather than a direct proxy for the equilibrium glass transition temperature.

The intermediate-to-high-temperature tan *δ* peaks appear at approximately 46.1 °C for the 14% SSC sample and 39.2 °C for the 26% SSC sample. Correlating these results with the DSC observations (Table 3), which show apparent melting endotherms of polyether diol crystalline domains in the range of approximately 38.7–53.2 °C, the high-temperature damping peak is more appropriately attributed to the melting of soft-segment crystals and the concomitant disruption of physical crosslinking networks, rather than to a glass transition of the hard or soft phases. During this thermal event, the melting of polyether diol crystallites releases chain segments from topological constraints, while the progressive weakening of hydrogen-bonded associations, particularly within the hard domains, reduces the effective physical crosslink density. These cooperative processes markedly enhance segmental mobility and internal friction, as reflected by the tan *δ* maximum, and are simultaneously accompanied by a precipitous decline in *E*′, signaling the collapse of the load-bearing hard-segment skeleton.

Collectively, the integrated DSC and DMA analyses provide a coherent picture of the SSC-dependent microstructural evolution. Increasing the SSC (1) depresses the storage modulus across all temperatures, indicating a transition from hard-segment-dominated rigidity to soft-segment-dominated compliance; (2) enhances the low-temperature relaxation intensity, confirming improved polyether diol segmental mobility; and (3) intensifies the intermediate-temperature damping response, which correlates with the melting of soft-segment crystals and the thermal disruption of physical crosslinks. These findings underscore that SSC serves as a decisive molecular lever for tuning the microphase-separated architecture—from a hard-segment-continuous rigid network to a soft-segment-continuous compliant matrix—thereby governing the material’s viscoelastic behavior and establishing the foundation for its balanced mechanical performance, including elastic recovery, energy dissipation, and fracture resistance, which are central to the high-performance pavement application targeted in this study.

### 4.2. Rheological Behavior and Workability

#### 4.2.1. Influence of SSC on Viscosity

**Figure 8:**
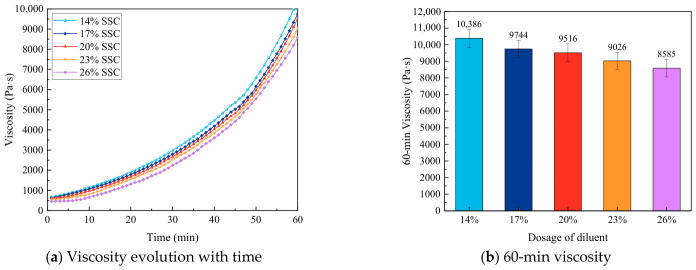
Viscosity of PU-PUa with different SSC.

The rheological behavior of the PU-PUa system, specifically the influence of SSC on viscosity, is illustrated in Figure 8. The nascent viscosity of the binder is mainly governed by the intrinsic viscosity of the precursors and their instantaneous intermolecular associations. The initial viscosity for all formulations exceeded 500 mPa·s. This relatively high starting point is attributed to the cyclic structure of the isocyanate and the secondary amine structure of the polyaspartic ester, coupled with the intense hierarchical hydrogen bonding and dipole–dipole interactions between these highly polar species.

During the polymerization process, a progressive increase in viscosity was observed across all groups. At a constant diluent content of 4.5%, the 60-min viscosity values with SSC of 14%, 17%, 20%, 23%, and 26% were recorded as 10,389, 9744, 9552, 9026, and 8585 mPa·s, respectively. This inverse relationship between SSC and viscosity indicates that the long-chain, flexible polyether diols function as an effective internal plasticizer. Increasing the SSC effectively lowers the relative concentration of the high-viscosity hard-segment precursors, thereby attenuating the density of urea-urea hydrogen bonds. Furthermore, although the ether oxygen atoms in the soft segments could engage in hydrogen bonding with the hard segments, these interactions are significantly weaker than the bidentate urea associations. Therefore, the proliferation of soft segments physically disrupts the formation of rigid physical crosslinking networks, leading to a more fluid rheological state.

#### 4.2.2. Influence of Diluent on Viscosity

**Figure 9:**
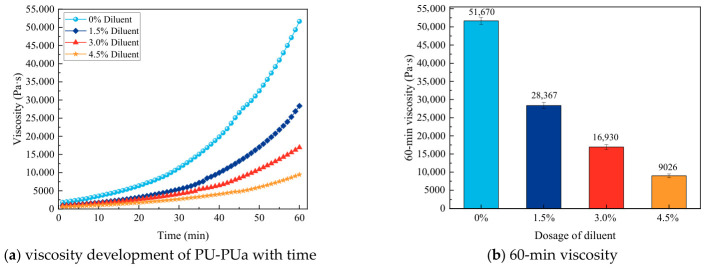
Viscosity of PU-PUa with various diluent contents.

The impact of the external diluent on the rheology of PU-PUa system is further detailed in Figure 9. While the initial viscosities remained comparable across various diluent dosages, a dramatic divergence in the viscosity profiles emerged as the reaction progressed. At the 60-min mark, the viscosity with diluent dosages of 0%, 1.5%, 3.0%, and 4.5% were 51,670 mPa·s, 28,367 mPa·s, 16,930 mPa·s, and 9026 mPa·s, respectively. Compared to the system without diluent, the viscosity of the systems with 1.5%, 3.0%, and 4.5% diluent were reduced by approximately 45.1%, 67.2%, and 82.5%, respectively. This demonstrated that the diluent was the most direct and effective means of regulating the viscosity, particularly by suppressing the rapid viscosity increased during the later stages, thereby significantly improving the workability of PU-PUa. This effect occurred because the diluent molecules intercalate between the growing polymer chains could increase the average intermolecular distance and provide a “screening effect” that effectively weakens the strong polar interactions. By systematically tailoring the SSC and diluent concentrations, the PU-PUa binder achieves an adjustable workability that accommodates the critical operational requirements of pavement construction, including mixing, hauling, and compaction process, thereby ensuring superior structural density and engineering quality.

### 4.3. Curing Characteristic and Temperature Sensitivity

#### 4.3.1. Influence of SSC on Curing Characteristic

**Figure 10:**
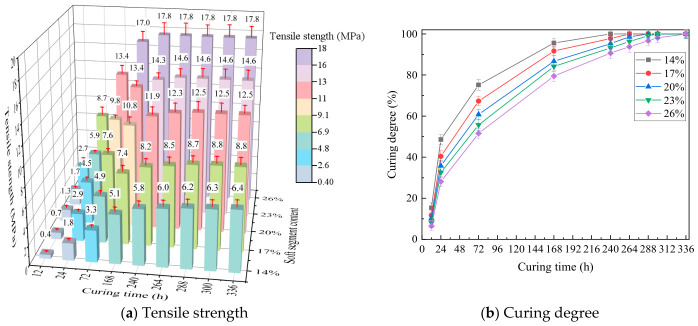
Strength development of PU-PUa with curing time.

The strength development profile of the PU-PUa system at 25 °C as a function of SSC and curing time is presented in Figure 10. Curing time was defined as the time required for the tensile strength of PU-PUa specimens cured at 25 °C to reach its final stable value. It can be seen clearly that the SSC is a decisive factor in the curing kinetics of the PU-PUa system. Formulations with lower SSC exhibited markedly accelerated early-stage strength gain. Specifically, after 24 h of curing, the system with 14% SSC achieved 48.3% of its ultimate strength, whereas the system with 26% SSC reached only 28.1%, a nearly 1.7-fold difference in the growth rate. This disparity persisted through 72 h, with 14% SSC, the system maintains a substantially higher degree of development (73.2%) compared to the 26% system (48.6%). This indicates that a lower SSC usually implies a higher relative concentration of high-reactivity isocyanate and polyaspartic ester precursors per unit volume. This increases the collision frequency of functional groups and accelerates the in situ formation of a rigid urea/urethane-rich skeleton. Consequently, the system with 14% SSC reached the full strength at approximately 264 h, while the 26% system required 336 h at the same curing condition. These results suggest that tailoring the SSC is a highly effective strategy for enhancing early-age mechanical performance and compressing the curing process, which is essential for rapid-repair pavement applications.

#### 4.3.2. Influence of Temperature on Curing Characteristics

**Figure 11:**
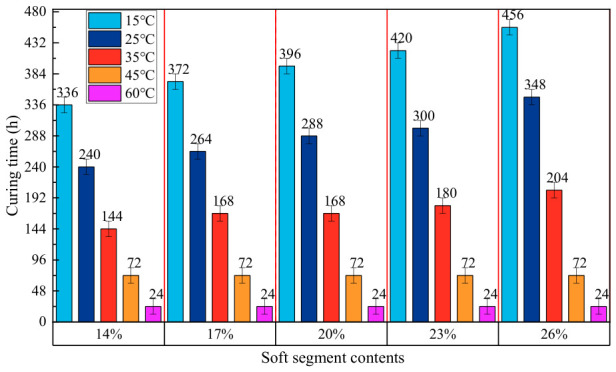
Curing time of PU-PUa at various temperatures.

The synergistic influence of curing temperature and SSC on the overall curing cycle is further elucidated in Figure 11. It is distinct that increasing the curing temperature significantly reduced the curing time for all systems. However, the degree of this reduction varied considerably with different SSC. For the system with 20% SSC, elevating the curing temperature from 15 °C to 60 °C resulted in a dramatic 94% reduction in the total curing time (from 396 h to 24 h). This exponential acceleration is attributed to the enhanced thermal motion and increased effective collisions between reactive groups (-NCO and -NH/-OH), which concurrently promotes the ordering kinetics of the hard-segment microdomains within the microphase separation structure.

Furthermore, the sensitivity of the curing rate to SSC was temperature-dependent. In the low-to-moderate temperature range (15–25 °C), the curing time for the system with 14% SSC decreased by 28.6% (from 336 h to 240 h), while the 26% system exhibited a 23.7% reduction. In this regime, the process is jointly governed by the chemical reaction rate and the diffusion-limited migration of molecular chains.

However, as the temperature increased further, this difference became more pronounced, in which a “kinetic convergence” was observed. In the high-temperature range from 45 °C to 60 °C, the curing time for the 14% system shortened from 72 h to 24 h, a reduction of 66.7%, while the curing time for the 26% system also decreased from 72 h to 24 h, exhibiting the same reduction rate. At these elevated thermal states, the reaction kinetics become dominated by the rapid polyaddition rate, which effectively masks the steric and mobility barriers associated with different soft-segment lengths.

Mastering these relationships between SSC, temperature, and strength evolution provides a scientific basis for optimizing mix designs and regulating curing protocols of the PU-PUa system. By dynamically adjusting construction plans according to environmental conditions, the curing duration can be significantly shortened, ensuring the rapid opening of resilient pavements to traffic.

### 4.4. Surface Wettability and Hydrophobicity

**Figure 12:**
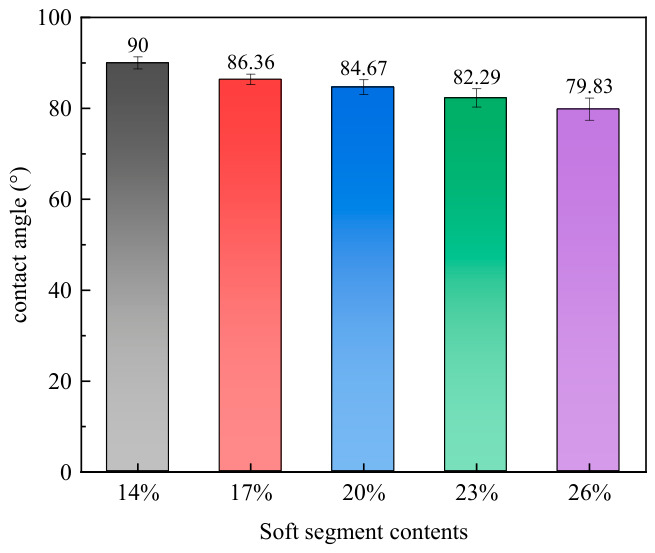
Contact angles of different SSC PU-PUa.

The surface characteristics of the PU-PUa binder, as quantified by static water contact angles, are presented in Figure 12. A clear inverse relationship was observed between the SSC and water contact angle. As the SSC increased from 14% to 26%, the water contact angle transitioned from a near-hydrophobic threshold of 90.0° down to 79.8°, 86.4°, 84.7°, 82.3°, and 79.8°, respectively. This trend indicates that higher SSC progressively enhances the surface wettability while compromising the intrinsic hydrophobicity of the material.

The observed variation in water contact angle is fundamentally attributed to the thermodynamically driven surface reconstruction of the segmented copolymer. The hard segments (comprising isocyanate, polyaspartic ester, and BDO) are characterized by high cohesive energy density due to the dense network of urea and urethane linkages. From a thermodynamic perspective, these highly polar domains tend to migrate toward the bulk interior to minimize the interfacial energy at the water–air interface. Conversely, the polyether-based soft segments, characterized by lower polarity and higher chain mobility, preferentially migrate and accumulate at the polymer surface.

As the SSC increases in the polymer system, the surface enrichment of polyether chains becomes more pronounced. Although the alkane backbones in the soft segments are relatively non-polar, the increasing density of ether oxygen atoms, which can act as hydrogen-bond acceptors for water molecules elevates the surface free energy of the binder. Thereby, the surface of the polymer becomes more hydrophilic, leading to the observed reduction in contact angle.

In pavement engineering, the moisture susceptibility of the binder is a critical determinant of long-term structural durability. A higher contact angle (as seen in the formulations with a low SSC) facilitates the formation of a solid hydrophobic barrier on the aggregate surface. This barrier effectively repels moisture infiltration and suppresses capillary suction within the mixture’s pore structure [22], and thus mitigating potential stripping and moisture-induced damage. Accordingly, optimizing the SSC to maintain a superior hydrophobic state is essential for ensuring the moisture stability of PU-PUa-based binder for pavement materials.

### 4.5. Interfacial Adhesion to Aggregate

#### 4.5.1. Bonding Strength

**Figure 13:**
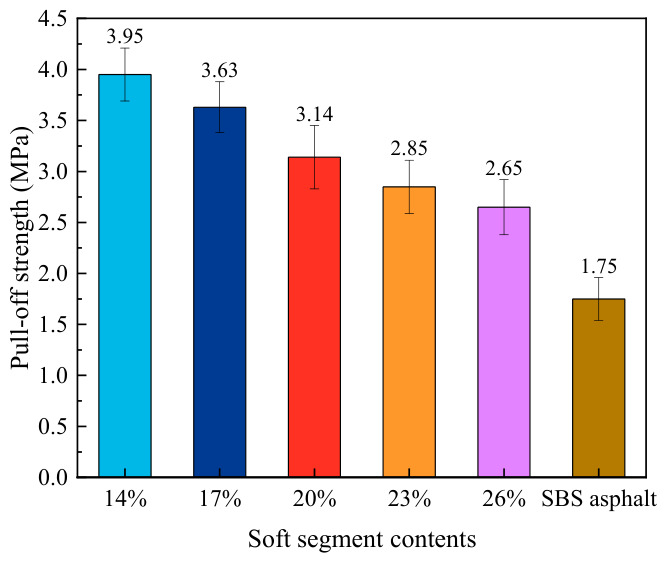
Results from pull-off tests.

The interfacial adhesion performance of the PU-PUa binder, as evaluated by pull-off tests on basalt substrates, is illustrated in Figure 13. A negative correlation was observed between the SSC and the pull-off strength. As the SSC increased from 14% to 26%, the pull-off strength systematically decreased from 3.95 MPa to 2.65 MPa. Even at the highest SSC (26%), the binder’s adhesion strength (2.65 MPa) remained much higher than the typical value of conventional SBS-modified asphalt (1.75 MPa), underscoring the superior bonding capability of the PU-PUa system.

The observed degradation in pull-off resistance with increasing SSC is primarily rooted in the structural attenuation of the hierarchical hydrogen-bonding network. The urea bonds (-NHCONH-), formed through the precise reaction of isocyanates and amine-functionalized polyaspartic esters, constitute the high-polarity hard segments [23,24]. These segments drive the formation of rigid microdomains and provide the requisite cohesive strength through dense, bidentate hydrogen bonding. As the SSC increases, the spatial density of these urea-urea associations is effectively diluted, leading to a reduction in the physical crosslinking density of the hard-segment skeleton.

While secondary hydrogen bonds between hard and soft segments (e.g., N–H···O=C or N–H···O-ether) persist, their bond energies are significantly lower than those of the primary urea-urea interactions. As a result, the increasement of soft segments cannot thermodynamically compensate for the loss of cohesion caused by the fragmentation of the hard-segment domains. In pavement applications, this reduction in cohesive integrity manifests macroscopically as a decline in pull-off strength. However, the interfacial bond maintained by the remaining polar groups could still ensure an adhesion strength consistently exceeding 2.0 MPa. This exceptional bonding performance is a critical factor in enhancing the raveling resistance of the mixture and ensuring the long-term durability of the pavement under heavy traffic loads and environmental erosions.

#### 4.5.2. Adhesion State of Polymer Binders to Aggregate After Water Erosion

**Figure 14:**
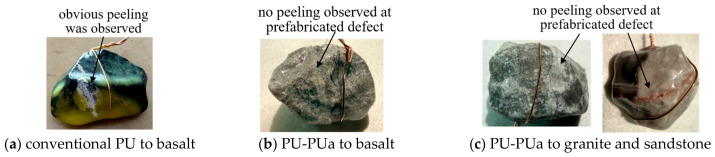
Adhesion state of polymer binders to aggregate.

Figure 14 illustrates the adhesion state of polymer binders to aggregate surfaces after water erosion. The PU-PUa binder exhibited exceptional adhesion to the aggregate. After immersion in boiling water at 100 °C for 10 min, no visible interfacial deterioration was observed between the PU-PUa film and the aggregate. The film surface remained smooth, and no obvious peeling or blistering occurred even at the prefabricated copper-wire defect (Figure 14b,c), indicating that the hierarchical hydrogen-bonding network and the intrinsic hydrophobicity of PU-PUa effectively suppress water infiltration along the interface.

In contrast, the interface between conventional PU and the aggregate shown in Figure 14a changed noticeably after boiling-water exposure, with bubbles and peeling appearing at the prefabricated defect of the film, suggesting that the hydrothermal environment weakened its interfacial stability. Accordingly, compared with conventional PU, PU-PUa maintains a more stable interfacial structure and higher debonding resistance after boiling-water exposure, confirming its superior adhesion to aggregates. In addition, whether for acidic (granite and sandstone) or alkaline (basalt) aggregates, PU-PUa exhibits excellent aggregate adhesion, which is significantly superior to that of asphalt-based materials.

### 4.6. Mechanical Performances

#### 4.6.1. Hardness

**Figure 15:**
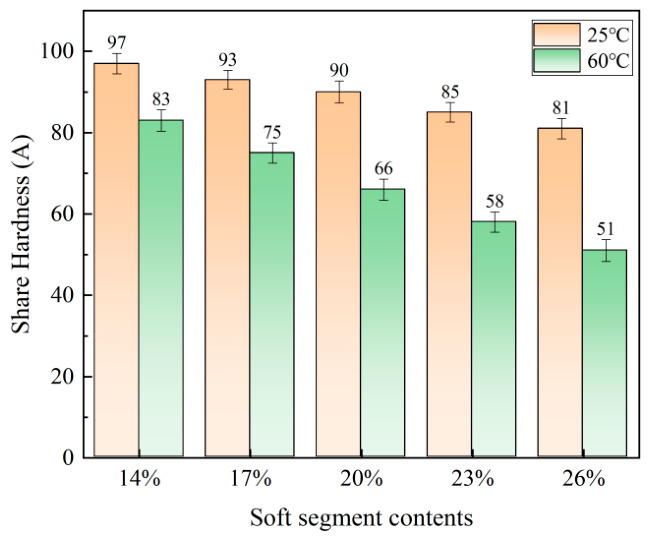
Hardness of PU-PUa.

The Shore A hardness of the PU-PUa binder as a function of SSC is presented in Figure 15. The results reveal a pronounced sensitivity of surface hardness to the segmented composition. As the SSC increased from 14% to 26%, the hardness at 25 °C decreased from 97 to 81, representing a 16.5% reduction, while at 60 °C it decreased from 83 to 51, representing a 38.6% reduction in indentation resistance. This trend is intrinsically governed by the spatial density of the rigid physical crosslinking network. The hard segments, characterized by dense urea-urea hydrogen bonding, act as a framework that provides the material with its requisite stiffness and resistance to localized deformation. As the SSC increases, the volume fraction of these rigid domains is effectively diluted, leading to an attenuation of the physical crosslinking density. Furthermore, elevated temperature exacerbates this softening by enhancing molecular chain mobility and weakening hydrogen bond interactions, resulting in a more pronounced hardness drop at 60 °C.

The evolution of hardness serves as a primary indicator of the material’s abrasion resistance and long-term surface durability [25,26]. In pavement engineering, the dominant damage modes are fatigue wear induced by repeated traffic loading and abrasive wear caused by hard particles; under such conditions, a certain level of hardness is advantageous. However, excessively high hardness—associated with very low SSC—tends to induce brittle fracture and poor impact resistance, while excessively low hardness—arising from high SSC—may compromise structural support and accelerate wear. Therefore, the optimal wear performance likely resides in an intermediate range of SSC, where the hard-segment microdomains provide effective frictional energy dissipation barriers while the soft-segment matrix ensures elastic recovery and prevents catastrophic surface failure. From a microscopic perspective, the hard-segment microdomains serve as the primary barrier against frictional energy dissipation. As this barrier is weakened by higher SSC, the elastic recovery of the soft segment matrix may be insufficient to fully compensate for the loss of structural rigidity, potentially accelerating the wear process.

In pavement engineering, the selection of SSC should be strategically tailored to the specific service environment. For heavy-duty traffic sections requiring high-modulus characteristics, a lower SSC formulation (e.g., 14–17%) is recommended to ensure structural longevity. Conversely, for pavement structures subjected to significant thermal fluctuations or requiring enhanced strain accommodation, a moderately higher SSC can be utilized to optimize the balance between flexibility and surface protection.

#### 4.6.2. Tensile Properties

**Figure 16:**
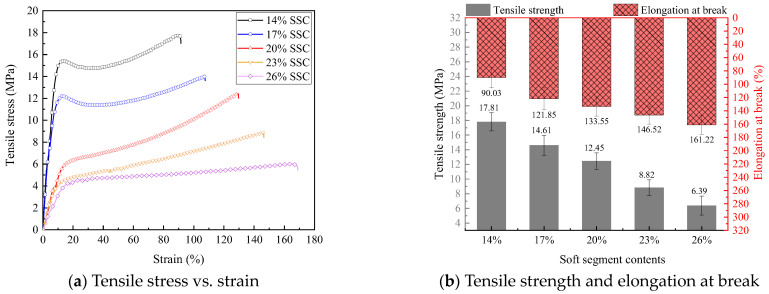
Tensile properties of PU-PUa.

The mechanical behavior of the PU-PUa binder was analyzed through tensile tests. The typical stress–strain curves and the evolution of mechanical performances (tensile strength and elongation at break) as a function of SSC are illustrated in Figure 16.

The experimental results demonstrate a significant regulatory effect of the SSC on the mechanical response of the PU-PUa system. As shown in Figure 16a, the stress–strain profiles undergo a distinct transition from a high-modulus, quasi-brittle behavior to a compliant, high-ductility response with increasing SSC. Quantitatively, as the SSC increased from 14% to 26%, the tensile strength markedly decreased from 17.81 MPa to 6.39 MPa (a 64.4% reduction), while the elongation at break systematically rose from 90.03% to 161.22% (a 78.9% increase).

Statistical analysis reveals a linear correlation within the investigated range: for every 1% increase in SSC, the tensile strength declines by approximately 0.96 MPa, whereas the elongation at break gains an average of 5.81 percentage points. This inverse relationship highlights the classic strength-ductility balance inherent in segmented copolymers, where the material can be precisely tailored to meet specific pavement requirements, ranging from high-strength structural reinforcement to high-toughness deformation accommodation.

This regular variation arose from the reconstruction of the microstructure. As the SSC increased, the proportion of hard segments composed of isocyanates and amine-based chain extenders correspondingly decreased, resulting in a reduced density of hard segment microstructure that formed the physical crosslinking network. Specifically, the dilution of the strong hydrogen bond network between urea bonds within the hard segment micro-structure directly weakened the load-bearing capacity. Meanwhile, the continuous soft segment phase provided greater freedom for molecular chain movement, enabling the material to undergo more significant plastic deformation after yielding and thereby exhibiting higher elongation at break. By precisely regulating the SSC, the material could be tailored from high strength with low elongation to low strength with high toughness.

When PU-PUa was used as a pavement material to replace asphalt or other polymer binders, it must withstand millions of repetitive loads during service. The exceptional fatigue performance stemmed from the synergistic effect of strength and toughness [27,28], which could enable the material to efficiently dissipate energy and delay micro-crack initiation and propagation under cyclic loading, potentially endowing the pavement with outstanding crack resistance and fatigue durability. Direct confirmation of this potential, however, required dedicated fatigue testing under cyclic loading conditions, which was a critical focus for the subsequent research.

#### 4.6.3. Resilient Modulus

**Figure 17:**
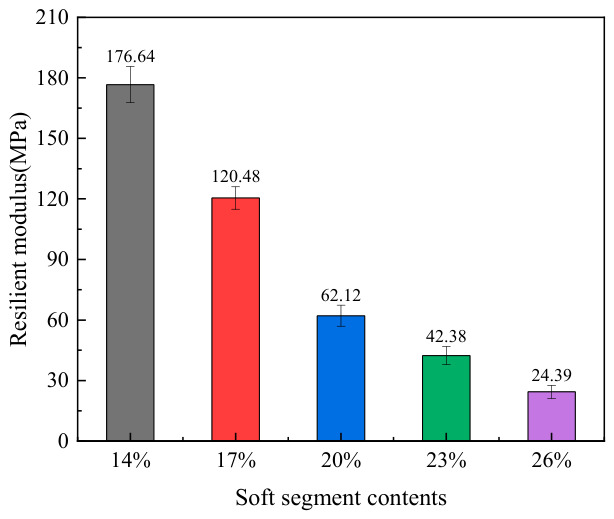
Resilient modulus of PU-PUa.

The resilient modulus of PU-PUa with varying SSC were presented in Figure 17. As the SSC increased from 14% to 26%, the elastic modulus monotonically decreased from 176.64 MPa to 24.39 MPa, representing a reduction of 86.2%, with the most pronounced decline (approximately 48.4%) occurring in the range of 17–20%. This trend was attributed to the structural transition from a hard-segment continuous phase to dispersed microdomains. At low SSC, the hard segments formed a percolating rigid skeleton through a dense hydrogen bonding network, endowing the material with a high modulus. With increasing SSC, the physical crosslinking density of the hard segments decreased, and the soft segment continuous phase gradually dominated the elastic response, leading to a sharp reduction in modulus.

The resilient modulus directly influenced the material’s rutting resistance and stress buffering capacity. High soft segment materials with low modulus were suitable for stress-absorbing layers or low-temperature crack-resistant scenarios, whereas low soft segment materials with high modulus were more appropriate for heavy-duty pavements.

#### 4.6.4. Microstructural Mechanism Analysis

The observed evolution of mechanical properties is intrinsically governed by the reconstruction of the microphase-separated morphology. The hard segments, which form nanoscale physical crosslinking nodes via bidentate urea–urea hydrogen bonds, dictate the initial modulus and ultimate tensile strength of the system. As the SSC increases, the volumetric fraction of these rigid domains is progressively diluted, leading to a marked reduction in the density of the physical network. In particular, the attenuation of urea–urea associations—the strongest hydrogen bonds within the system—lowers the energy barrier for interchain slippage, thereby accounting for the observed decline in tensile strength.

Conversely, the systematic increase in elongation at break with rising SSC originates from two synergistic mechanisms: entropic elasticity and sacrificial bond dissipation. The continuous soft segment phase, comprising flexible polyether chains, offers enhanced conformational freedom, enabling substantial chain rearrangement and disentanglement after yielding. More importantly, the multi-scale hydrogen-bonding network (encompassing urethane–urea, urea–ether, and residual urethane–urethane interactions) functions as a hierarchy of reversible sacrificial bonds. Under high tensile strain, these weaker non-covalent linkages preferentially rupture prior to covalent bond scission, dissipating mechanical energy and shielding the polymer backbone from premature failure. This progressive, energy-dissipative mechanism effectively delays micro-crack initiation and propagation, endowing the PU-PUa system with a unique combination of strength and ductility that is highly desirable for pavement applications.

### 4.7. Comparison with Conventional PU and Asphalt Binder

To benchmark the developed PU-PUa binder against existing pavement materials, its key properties were compared with conventional PU binders and typical SBS-modified asphalt, as summarized in Table 4. The data for PU and SBS-modified asphalt binders are compiled from typical values in representative studies. The PU-PUa data originate from the present study (SSC ranging from 14% to 26%).

PU-PUa achieves tunable tensile strength (6–18 MPa) and elongation (90–160% at 25 °C) with the variation of SSC, higher than the typical SBS-modified asphalt values reported in the cited literature (1–2.5 MPa). Its pull-off strength (2.6–4.0 MPa) is also significantly higher than that of asphalt (0.6–1.5 MPa) and falls within the upper range of the reported PU binder values. By adjusting SSC (14–26%), both strength and ductility can be tailored—a unique design flexibility. Moreover, its water contact angle (80–90°) can be increased by reducing SSC to enhance hydrophobicity and moisture resistance. In addition, PU-PUa is a cold-mix, cold-cure system with low initial viscosity (500–1000 mPa·s at 25 °C), enabling easy mixing and compaction at ambient temperature. Curing time (240–336 h at 25 °C) can be shortened to 24 h at 60 °C, ideal for rapid repair. Thanks to its hierarchical hydrogen-bonding network and nanoscale microphase separation, PU-PUa offers superior aging and hydrolysis resistance compared to conventional PU, while asphalt suffers from UV/thermal degradation.

Overall, PU-PUa bridges high-strength thermosets and flexible elastomers, showing favorable values compared with the literature-reported ranges for conventional asphalt binders in adhesion, strength, and moisture resistance while offering broader tunability than typical PU binders. These advantages, rooted in the synergistic design of multi-scale hydrogen bonding and microphase separation, position PU-PUa as a next-generation binder for resilient and sustainable pavements.

It should be noted that the present study did not include dedicated long-term durability tests, such as cyclic fatigue, UV-weathering, thermo-oxidative aging, and freeze–thaw tests. Therefore, the discussion of fatigue resistance, aging resistance, and long-term pavement durability should be regarded as potential implications inferred from the observed mechanical performance, interfacial adhesion, and short-term hydrothermal stability, rather than as direct experimental confirmation. Further durability-oriented testing is required to quantitatively evaluate the long-term service performance of the PU-PUa binder under realistic pavement conditions.

## 5. Conclusions

This study synthesized PU-PUa binder through a one-step method and investigated the influence of soft segment content (SSC) on curing behavior, interfacial properties, and mechanical performance. The FTIR and AFM results support the formation of urethane/urea structures, hydrogen-bonding interactions, and surface morphological heterogeneity, while the DSC and DMA results reveal the effects of SSC on soft-segment mobility, crystallization/melting behavior, and temperature-dependent mechanical relaxation. These findings provide a basis for interpreting the structure-property relationships of the PU-PUa binder. The main conclusions are as follows:

(1) The combination of aliphatic isocyanate and polyaspartic ester introduced urea-rich hard segments and hydrogen-bonding interactions into the PU-PUa network, forming hard-segment-rich domains that act as physical crosslinking points. Meanwhile, the flexible soft segments of polyether diol provide chain mobility and energy dissipation. DSC and DMA results further confirmed that increasing SSC enhanced soft-segment mobility, promoted soft-segment crystallization/reorganization, and reduced the storage modulus, indicating a stronger contribution of the soft phase to viscoelastic relaxation and toughness.

(2) The workability and curing behavior of the PU-PUa system can be precisely controlled by adjusting the SSC and curing temperature. Increasing the SSC or adding a small amount of diluent significantly reduces viscosity, whereas decreasing the SSC or raising the temperature accelerates strength development and shortens the curing cycle. This adaptability provides a broad operational window for critical processes such as mixing, hauling, paving, and compaction, ensuring both construction quality and project efficiency.

(3) The surface wettability and interfacial adhesion of the PU-PUa binder are critically governed by the surface reconstruction of soft segment. Lowering the SSC enhances the intrinsic hydrophobicity, creating an effective moisture barrier on aggregate surfaces. Furthermore, the material maintains exceptional interfacial bonding states and Shore hardness, ensuring superior stripping resistance and long-term abrasion durability under the combined effects of moisture and repetitive traffic loading.

(4) The PU-PUa binder exhibits an outstanding strength-ductility combination, with tensile strengths ranging from 6.0 MPa and 18.0 MPa and elongations at break from 90% to 160%. The rigid hard segment skeleton ensures high load-bearing capacity, while the synergistic toughening effect, enabled by the sacrificial bond mechanism, effectively retards micro-crack initiation and propagation. These properties suggest that the PU-PUa system may have potential advantages in crack-resistance applications. However, its fatigue durability and long-term pavement performance require further verification through dedicated cyclic loading, aging, UV-weathering, thermo-oxidative aging, and freeze–thaw tests.

(5) At the binder level, an SSC range of approximately 17–20% is recommended as a balanced formulation window. This range maintains relatively high bonding strength, Shore hardness, tensile strength, and modulus while improving flexibility and workability compared with the lowest-SSC formulation. However, because mixture-level tests were not conducted, this range should be regarded as a binder-level recommendation rather than a final optimum for pavement mixtures.

## Figures and Tables

**Table 1:** Fundamental properties of raw materials.

Properties	Units	Test Results		
		Isocyanate	Polyether Diol	Polyaspartic Ester
Mass fraction of -NCO	%	22.05	N/A	N/A
Functionality	N/A	3	2	2
Equivalent weight	g/mol	173	580	236
Molecular weight	Mn	573	1160	472
Viscosity (25 °C)	mPa·s	1750	570	1250
Density	g/cm^3^	1.051	0.986	1.033
Color	APHA	≤40	≤50	≤230
Melting point	°C	−67	25	10
Flash point	°C	160	280	310

**Table 2:** Equivalent weight-based formulation ratios for PU-PUa (mol).

Group	Soft Segment Content (SSC)	Isocyanate (NCO)	Polyaspartic Ester (NH)	Polyether Diol (OH)	BDO (OH)
PU-PUa-I	14%	9.97	7.81	1.00	1.11
PU-PUa-II	17%	7.85	5.30	1.00	1.54
PU-PUa-III	20%	6.87	4.43	1.00	1.39
PU-PUa-IV	23%	5.89	3.84	1.00	1.01
PU-PUa-V	26%	4.71	3.31	1.00	0.40

**Table 3:** DSC-derived thermal transition parameters.

Group	SSC	*T*_g,s_ (°C)	*T*_cc_ (°C)	Δ*H*_cc_ (J/g)	*T*_m_ (°C)	Δ*H*_m_ (J/g)
PU-PUa-I	14%	−39.4	−2.8	0.089	38.7	2.119
PU-PUa-V	26%	−51.6	−5.9	0.173	53.2	0.414

**Table 4:** Comparison of PU-PUa, conventional PU, and SBS-modified asphalt.

Property	PU-PUa	Conventional PU [1,2,7,15,22,23,29,30,31]	SBS-Modified Asphalt [2,31,32,33]
Curing time (h, @25 °C)	240–336	57–360 [2,15]	N/A (Thermoplastic)
Viscosity (mPa·s)	500–1000 (@25 °C)	500–4500 [15,22,23,31] (@25 °C)	2000–5000 (@135 °C) [32,33]
Water contact angle (°)	80–90	50–110 [22,23]	70–95 [31,32,33]
Bonding strength (MPa)	2.6–4.0	1.2–3.0 [1,2,15,30]	0.6–1.5 [2]
Adhesion post-erosion	Intact	Severe peeling	Intact (Acid-sensitive)
Shore A hardness	81–97	70–94 [2,7,30]	N/A (very soft)
Tensile strength (MPa)	6.0–18.0	3.5–15.0 [1,2,7,15,22,23,29,30]	1.0–2.5
Elongation at break (%)	90–160	30–170 [1,2,7,15,23,29,30]	150–300 (@25 °C), <50 (@5 °C)

Note: The values for conventional PU binders and SBS-modified asphalt were collected from different literature sources and are presented as typical reported ranges. Because the testing methods, temperatures, substrates, curing conditions, and aging states varied among the cited studies, the comparison is intended only as a literature-based benchmark rather than a direct one-to-one performance ranking. For SBS-modified asphalt, the tensile strength data were measured in this study, while the remaining values were collected from the cited literature.

## Data Availability

The raw data supporting the conclusions of this article will be made available by the authors on request.
